# Elevated C-peptide and insulin predict increased risk of colorectal adenomas in normal mucosa

**DOI:** 10.1186/1471-2407-12-389

**Published:** 2012-09-05

**Authors:** Adriana C Vidal, Pauline Kay Lund, Cathrine Hoyo, Joseph Galanko, Lauren Burcal, Rachel Holston, Berri Massa, Oluwaseun Omofoye, Robert S Sandler, Temitope O Keku

**Affiliations:** 1 Department of Obstetrics and Gynecology, and Program of Cancer Detection, Prevention and Control, for Duke University School of Medicine, Durham, North Carolina; 2 Department of Medicine and Center for Gastrointestinal Biology & Disease School of Medicine, University of North Carolina, Chapel Hill, North Carolina; 3 Department of Cell and Molecular Physiology, School of Medicine, University of North Carolina, Chapel Hill, North Carolina

**Keywords:** Insulin, C-peptide, Insulin-like growth factor binding protein

## Abstract

**Background:**

Lower concentrations of the insulin–like growth factor binding protein-1 (IGFBP-1) and elevated concentrations of insulin or C-peptide have been associated with an increase in colorectal cancer risk (CRC). However few studies have evaluated IGFBP-1 and C-peptide in relation to adenomatous polyps, the only known precursor for CRC.

**Methods:**

Between November 2001 and December 2002, we examined associations between circulating concentrations of insulin, C-peptide, IGFBP-1 and apoptosis among 190 individuals with one or more adenomatous polyps and 488 with no adenomatous polyps using logistic regression models.

**Results:**

Individuals with the highest concentrations of C-peptide were more likely to have adenomas (OR = 2.2, 95% CI 1.4-4.0) than those with the lowest concentrations; associations that appeared to be stronger in men (OR = 4.4, 95% CI 1.7-10.9) than women. Individuals with high insulin concentrations also had a higher risk of adenomas (OR = 3.5, 95% CI 1.7-7.4), whereas higher levels of IGFBP-1 were associated with a reduced risk of adenomas in men only (OR = 0.3, 95% CI 0.1-0.7). Overweight and obese individuals with higher C-peptide levels (>1^st^ Q) were at increased risk for lower apoptosis index (OR = 2.5, 95% CI 0.9-7.1), an association that remained strong in overweight and obese men (OR = 6.3, 95% CI 1.0-36.7). Higher levels of IGFBP-1 in overweight and obese individuals were associated with a reduced risk of low apoptosis (OR = 0.3, 95% CI 0.1-1.0).

**Conclusions:**

Associations between these peptides and the apoptosis index in overweight and obese individuals, suggest that the mechanism by which C-peptide could induce adenomas may include its anti-apoptotic properties. This study suggests that hyperinsulinemia and IGF hormones predict adenoma risk, and that outcomes associated with colorectal carcinogenesis maybe modified by gender.

## Background

Colorectal adenomas are precursors to colorectal cancer (CRC) [1], and CRC is the second most common cancer in men and women worldwide [2]. Reducing CRC risk will depend on identification of potential markers of precancerous adenomas. Components of the insulin-IGF axis have been key targets to identify potential biomarkers of adenoma risk, in part because western diets, physical inactivity and other factors associated with obesity, insulin resistance and diabetes history, are known risk factors for colon cancer [3]. The mechanisms by which obesity and insulin resistance promote CRC are not well understood, although insulin is a crucial component in the regulation of energy metabolism. Elevated circulating insulin levels have also been associated with mitogenic [4,5] and anti-apoptotic effects in overweight and obese individuals [6]. Thus, higher circulating insulin levels may promote colorectal adenomas and cancer through increased proliferation or reduced apoptosis [5,7].

Insulin, insulin growth factor I (IGF-I) and IGF-II are structurally related proteins that share common receptors [8]. At high concentrations, insulin may utilize insulin receptors (IR), IGF1 receptors (IGF1R) or hybrid IR/IGF1R to regulate cell proliferation and apoptosis [9-11]. Insulin can also act directly to promote IGF-I biosynthesis or can enhance IGF-I bioavailability by inhibiting the production of IGF-binding proteins (IGFBP) such as IGFBP-1 [12,13]. IGFBP-1 plays an important role in glucose homeostasis, and circulating IGFBP-1 is inversely related to C-peptide and insulin levels [14,15]. Furthermore, IGFBP-1 can also induce apoptosis in breast and prostate cancer cells *in vitro* as well as suppress tumor growth in an insulin dependent and independent manner [16]. Thus, IGFBP-1 levels may influence colorectal adenomas and cancer development via two mechanisms: inhibition of the proliferative actions of insulin and IGF, and promotion of apoptosis. Disentangling these relationships will be important in determining the utility of these peptide hormones as disease markers.

We have previously reported a positive association between elevated fasting plasma insulin levels and adenomas in the Diet and Health Study III [17]. More recently, we observed that local expression of IGFBP-3 was inversely related to adenomas [18]. Based on these previous observations, we hypothesized that elevated IGFBP-1 is associated with reduced risk of adenomas and low apoptosis in normal mucosa. Few studies have evaluated IGFBP-1 and C-peptide or insulin in relation to colorectal adenomas and cancer and the results are inconsistent [19-24]. Inconsistencies may be due in part to differences in race/ethnicity [22] and gender [24]. Moreover, the relationships between these markers and apoptosis have not, to our knowledge, been addressed [17]. The aim of this study was to determine the associations of IGFBP-1, insulin, and C-peptide (a surrogate biomarker of pancreatic insulin secretion), with colorectal adenoma (adenomatous polyps) in the Diet and Health study IV, in a majority White cohort and whether these associations vary by gender.

## Methods

### Study population

Study participants in the Diet and Health Study IV were drawn from outpatients who underwent screening colonoscopy between November 2001 and December 2002 at the University of North Carolina hospitals (Chapel Hill, NC). Eligible subjects provided informed consent and agreed to participate in a telephone interview, give rectal biopsies or have blood drawn. Subjects were excluded for the following reasons, incomplete examination (cecum not reached), age <30 years, inability to give informed consent, polyposis (>100 polyps), previous colon resection or cancer, colitis, and previous colon adenoma. All histology and classification of colon polyps in the study were performed as previously described [17]. Advanced adenoma was defined as having an adenoma at least 1 cm in diameter, histology of villoglandular or villous or severe atypia. Individuals who had one or more adenomatous polyps were defined as cases while control subjects had no adenomatous polyps. The study was approved by the School of Medicine institutional review board at the University of North Carolina.

### Data collection

The data collection was similar to previous protocols [17,25,26]. Briefly, eligible and consenting participants provided information about the time of last meal, (to confirm an overnight fast) and the type of colonoscopy preparation used. We also measured height, body weight, and waist and hip circumference. A total of 1027 subjects were eligible, 123 (12%) refused to participate, 91 (9%) were not asked because the research assistant was not available, and an additional 107 (10%) patients were classified as ineligible after the colonoscopy due to incomplete or unsatisfactory preparation. The study was completed by 701 individuals, with a response rate of 76% (number interviewed/number eligible). Complete information on insulin, C-peptide, IGFBP-1, adenomas and apoptosis was available for 678 subjects. Enrolled subjects completed telephone interviews about diet and lifestyle within 12 weeks of colonoscopy. The lifestyle questionnaire was used to collect data about demographics, family history, education, medical history, physical activity, and other environmental factors. Dietary information was collected using the NCI Diet History food frequency questionnaire that queried foods and usual portion size (small, medium, or large) consumed [27].

### Biological specimens and laboratory assays

Specimens’ preparation and handling have been previously described [8]. Subjects used either a balanced electrolyte polyethylene glycol lavage or a phosphate-containing purge prep prior to colonoscopy. At the beginning of the endoscopic procedure, six mucosal pinch biopsies were obtained 8 to 10 cm from the anal verge using standard disposable, fenestrated colonoscopy forceps (Wilson-Cook, Winston-Salem, NC). The same site was sampled in all subjects. Blood samples were obtained prior to administration of medication through an i. v. catheter and processed within 2–6 hours in the lab. Samples were kept at 4^o^C prior to processing. Plasma from the blood samples was stored in aliquots at −80^o^C until assayed. Care was taken to avoid repeated freezing and thawing of samples. We analyzed samples from patients with excellent colonoscopy preparation and confirmed overnight fast (based on verbal response about last food intake and supportive evidence of a clean colon). Circulating insulin, C-peptide and IGFBP-1 levels were measured by ELISA using reagents from Diagnostic Systems Laboratory (Webster, TX). Laboratory personnel were blinded to the case or control status of samples. The intra-assay coefficient of variation was 2.6% for insulin, 4.2% for C-peptide and 5.1% for IGFBP-1. The inter-assay coefficient of variation was <12% for insulin, C-peptide and IGFBP-1 measures.

### Assays of apoptosis

Colonic biopsies were fixed in 10% buffered formalin and processed by routine histology. Apoptosis was scored by personnel who were trained by study pathologist to differentiate between apoptosis and necrosis. A subset of slides was re-scored by a second trained scorer (TOK) in a blinded fashion. The inter-rater level of agreement was >94%. We chose to use morphology to assess apoptosis in this study based on our previous experience. Previously, we have used two methods, morphology and TUNEL to assess apoptosis and found a high level of correlation between the two methods (r = 0.73, p = 0.01) [17].

### Statistical analysis

Comparison of continuous and categorical variables between adenoma cases and non-adenoma controls were made using t tests and Chi square tests, respectively. The relationships between plasma insulin, C-peptide, IGFBP-1 and colorectal adenomas were assessed in the study population by comparing mean values between case and control subjects using t tests. The distribution of insulin, C-peptide, IGFBP-1 measures among control subjects was used to generate quartile values. The lowest quartile of each measure was considered as the reference. Apoptosis was expressed as the average number of apoptotic cells per crypt. Using the median as cut point, we divided apoptosis measures into lower half (below the median) and upper half (above the median). Logistic regression models were used to examine the association between adenoma status and insulin, C-peptide, IGFBP-1 or apoptosis. Confounder candidates for inclusion in the model were age, race, sex, BMI, family history, total fat intake, alcohol intake, waist to hip ratio, diabetes history, smoking status, NSAID use, calcium intake and red meat intake. Each of these was added to a model containing the main variable of interest (in quartiles) and if at least two of the quartile parameter estimates changed by more than 10% then the variable was considered a potential confounder. All variables that met that standard were then entered into a backwards stepwise procedure with the main variable of interest being forced into the model.

In addition, we also performed the trend test to examine for linear increase over quartile 1 to quartile 4 (p-trend). The relationships between insulin, C-peptide, IGFBP-1 and BMI, and waist-to hip ratio (WHR) were assessed by the Spearman correlation coefficient. In cases, we also measured Spearman correlation coefficients between number and size of adenomas and C-peptide, insulin and IGFBP-1 levels.

## Results

**Figure 1:**
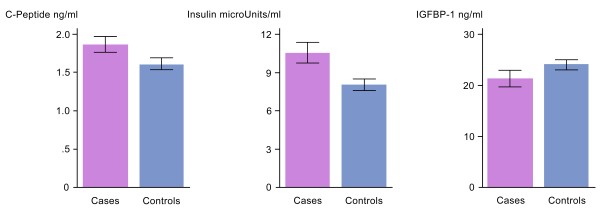
**Mean levels of circulating C-peptide, insulin and IGFBP-1 in adenoma cases and non-adenoma controls.** C-peptide (p = 0.05) and insulin (p = 0.008) levels were significantly elevated in cases compared to controls. Mean plasma IGFBP-1 was lower in cases than in controls but the results did not reach statistical significance.

The characteristics of study participants are shown in Table 1. Compared to controls (mean BMI = 27.1 se = 0.3), cases were significantly more likely to have higher BMI (mean BMI = 28.3 se = 0.4), and increased waist-hip ratio (WHR), mean WHR = 0.88 (se = 0.01) *vs.* mean WHR = 0.93 (se = 0.01). A higher proportion of cases reported increased consumption of alcohol and red meat intake (mean = 1.7 svgs/day se = 0.11) than controls (mean = 1.47 svgs/day se = 0.06). Apoptosis was also significantly lower in cases than controls (p = 0.0003) while the mean levels of circulating C-peptide (p = 0.05) and insulin (p = 0.008) were significantly elevated in cases than controls (Figure 1). Mean plasma IGFBP-1 was lower in cases than controls but the results did not reach statistical significance (Figure 1).

**Table 1:** Descriptive characteristics of adenoma cases and adenoma-free controls

**Variable**	**Cases (N = 190)**	**Controls (N = 488)**	**p-value**
Mean Age in Years (SE)	56.8 (0.7)	55.7 (0.5)	0.19
White (%)	156 (82)	383 (78)	0.34
Male (%)	112 (59)	188 (39)	0.0001
Mean Body Mass Index (SE)	28.3 (0.4)	27.1 (0.3)	0.02
Family History of CRC (%)	28 (17)	60 (14)	0.37
Mean Dietary Fat (g/day) (SE)	70.8 (2.7)	68.6 (1.5)	0.47
Mean Alcohol^1^ (SE)	12.6 (1.7)	8.2 (0.7)	0.02
Mean Waist/hip Ratio (SE)	0.930 (0.008)	0.886 (0.007)	0.0001
Smokers (%)	29 (17)	49 (11)	0.06
Mean Monthly NSAID Use in Past 5 Years (SE)	12.9 (2.0)	12.7 (1.4)	0.94
Mean Total Daily Calcium (mg) (SE)	744 (28)	785 (18)	0.23
Mean Red Meat (svgs/day) (SE)	1.73 (0.11)	1.47 (0.06)	0.03
Apoptosis (Morphology)^2^	3.33 (0.10)	3.76 (0.06)	0.0003
C-peptide (mean, se)	1.86 (0.10)	1.61 (0.08)	0.05
IGFBP-1 (mean, se)	21.3 (1.60)	24.1 (1.02)	0.13
Insulin (mean, se)	10.5 (0.8)	8.1 (0.5)	0.008
Number of Adenomas (mean, se)	1.58 (0.08)	-	-
Adenoma size (mean, se)^3^	6.21 (0.45)	-	

^1^ Daily grams from alcohol.

^2^Apoptosis – mean apoptotic cells per crypt.

^3^ Mean size of the largest adenoma.

Table 2 summarizes the associations between plasma insulin, C-peptide, IGFBP-1 and adenomas. Overall, elevated C-peptide or insulin showed a positive association with adenomas (p-trend ≤ 0.01). Compared to those with the lowest quartile (Q1) of plasma insulin or C-peptide levels, levels of these analytes in the fourth quartile (Q4) were associated with a 2 to 5-fold increased risk of adenomas (OR = 3.5, 95% CI 1.7-7.4, p-trend = 0.002, OR = 2.2, 95% CI 1.2-4.0, p-trend = 0.04, respectively). For C-peptide these associations may be stronger in men (Q4 OR = 4.4, 95% CI 1.7-10.9, p-trend =0.006), than women (OR = 1.6, 95% CI 0.7-4.0, p-trend = 0.58). Overall, associations were stronger in the 3^rd^ and 4^th^ Q, for both C-peptide and insulin (Table 2). On the other hand, we found a statistically significant association between higher levels of IGFBP-1 and reduced risk for adenoma in men (OR = 0.3, 95% CI 0.1-0.7, p-trend = 0.0007). These associations remained unaltered after excluding BMI and WHR from the statistical models.

**Table 2:** *Adjusted ORs and 95% CI for the relationship between the C-peptide, IGFBP-1 and adenoma status, overall, and among men and women

	**Overall**		**Men**		**Women**	
**Measure**	**Case/control (n)**	**Adjusted OR**^1^**(95% CI)**	**Case/control (n)**	**Adjusted OR**^1^**(95% CI)**	**Case/control (n)**	**Adjusted OR**^1^**(95% CI)**
**C-Peptide**						
Quartile 1	29/106	1.0 (Referent)	14/40	1.0 (Referent)	15/66	1.0 (Referent)
Quartile 2	32/108	1.2 (0.6, 2.1)	19/42	1.9 (0.7, 4.8)	13/66	0.8 (0.3, 2.1)
Quartile 3	40/101	1.4 (0.8, 2.6)	24/40	2.6 (1.0, 6.9)	16/61	1.0 (0.4, 2.4)
Quartile 4	71/105	2.2 (1.2, 4.0)	48/45	4.4 (1.7, 10.9)	23/60	1.6 (0.7, 4.0)
P trend		0.04		0.006		0.58
**IGFBP-1**						
Quartile 1	51/106	1.0 (Referent)	36/41	1.0 (Referent)	15/65	1.0 (Referent)
Quartile 2	52/103	0.9 (0.5, 1.6)	35/49	0.7 (0.3, 1.5)	17/54	1.2 (0.5, 3.0)
Quartile 3	37/105	0.7 (0.4, 1.2)	22/42	0.5 (0.2, 1.0)	15/63	1.3 (0.5, 3.1)
Quartile 4	29/105	0.6 (0.3, 1.1)	10/35	0.3 (0.1, 0.7)	19/70	1.4 (0.6, 3.4)
P trend		0.13		0.007		0.47
**Insulin**						
Quartile 1	21/88	1.0 (Referent)	14/37	1.0 (Referent)	7/51	1.0 (Referent)
Quartile 2	27/84	1.7 (0.8, 3.7)	17/32	1.3 (0.5, 3.5)	10/52	2.6 (0.7, 9.0)
Quartile 3	40/81	2.6 (1.2, 5.5)	29/31	3.1 (1.2, 7.9)	11/50	2.4 (0.7, 8.6)
Quartile 4	55/84	3.5 (1.7, 7.4)	31/33	2.8 (1.1, 7.1)	24/51	4.6 (1.5, 15.3)
P trend		0.002		0.02		0.03

*Adjusted for race, sex, BMI, WHR, diabetes history, alcohol intake, smoking and red meat intake.

We examined the relationship between apoptosis levels and C-peptide, insulin and IGFBP1 in males and females. We found no evidence for associations between levels of C-peptide, insulin and IGFBP-1, and the apoptosis index, among all individuals. However, restricting analyses for overweight and obese individuals, we found that higher C-peptide levels (>1^st^ Q) were associated with increased risk of lower apoptosis index (OR = 2.5, 95% CI 0.9-7.1, p-trend = 0.048), an association that persisted in men (OR = 6.3, 95% CI 1.0-36.7, p-trend = 0.04) (Table 3). Whereas higher levels of IGFB-1 in overweight and obese individuals overall were associated with a reduced risk of low apoptosis (OR = 0.3, 95% CI 0.1-1.0, p-trend = 0.10) (Table 3). Further restricting analyses by sex revealed no additional insights for IGFBP-1.

**Table 3:** *Adjusted ORs and 95% CI for the relationship between the C-peptide, IGFBP-1 and low apoptosis, among overweight and obese individuals (BMI > 30)

	**Overall**		**Men**		**Women**	
**Measure**	**Apoptosis (n, Lower/upper half)**	**Adjusted OR**^1^**(95% CI)**	**Apoptosis (n, Lower/upper half)**	**Adjusted OR**^1^**(95% CI)**	**Apoptosis (n, Lower/upper half)**	**Adjusted OR**^1^**(95% CI)**
**C-Peptide**						
Quartile 1	12/16	1.0 (Referent)	6/7	1.0 (Referent)	6/9	1.0 (Referent)
Quartile 2	26/20	3.1 (1.0, 9.5)	9/11	4.4 (0.7, 30.0)	17/9	3.9 (0.9, 16.1)
Quartile 3	33/24	2.7 (0.9, 8.1)	17/9	7.0 (1.1,45.3)	16/15	1.9 (0.5, 7.3)
Quartile 4	54/45	2.5 (0.9, 7.1)	30/21	6.3 (1.1, 36.7)	24/24	1.5 (0.4, 5.5)
P trend		0.0465		0.04		0.30
**IGFBP-1**						
Quartile 1	51/35	1.0 (Referent)	24/13	1.0 (Referent)	27/22	1.0 (Referent)
Quartile 2	44/33	0.9 (0.5, 1.9)	23/16	0.9 (0.3, 2.6)	21/17	1.0 (0.4, 2.5)
Quartile 3	22/19	0.9 (0.4, 2.1)	12/12	0.5 (0.1, 1.6)	10/7	1.4 (0.4, 4.9)
Quartile 4	8/18	0.3 (0.1, 1.0)	3/7	0.2 (0.03, 1.2)	5/11	0.4 (0.1, 1.5)
P trend		0.10		0.07		0.44
**Insulin**						
Quartile 1	11/14	1.0 (Referent)	6/8	1.0 (Referent)	5/6	1.0 (Referent)
Quartile 2	25/17	1.6 (0.5, 5.1)	16/5	2.3 (0.4, 13.1)	9/12	1.1 (0.2, 5.5)
Quartile 3	33/25	1.3 (0.4, 3.9)	17/13	0.7 (0.1, 3.4)	16/12	1.7 (0.3, 8.5)
Quartile 4	40/32	1.5 (0.5, 4.4)	17/10	1.0 (0.2, 4.9)	23/22	1.6 (0.4, 7.1)
P trend		0.46		0.99		0.53

*Adjusted for race, sex, alcohol intake, diabetes history, smoking and red meat intake.

We also examined the relationship between insulin, C-peptide, IGFBP-1, and obesity (BMI or waist-hip ratio (WHR), which are factors related to colorectal adenomas, CRC and insulin resistance (Table 4). In general, both insulin and C-peptide showed strong negative correlations with IGFBP-1. Both insulin and C-peptide showed highly significant positive correlations with BMI and WHR. In addition, there was strong inverse correlation between IGFBP-1 and BMI (r = − 0.49, p = 0.0001), and IGFBP-1 and WHR (r = −0.29, p = 0.0001) (Table 4). The associations for men and women were comparable. Among adenoma cases, no correlations were found for C-peptide, insulin or IGFBP-1 levels and either number of adenomas (r = 0.03, p = 0.65; r = 0.004, p = 0.96; r = 0.06, p = 0.41, respectively) or adenoma size (r = −0.05, p = 0.50; r = 0.14, p = 0.06; r = −0.02, p = 0.80, respectively).

**Table 4:** Correlations between insulin, C-peptide, IGFBP-1 and BMI or WHR

	**C-Pep**	**IGFBP-1**	**Insulin**	**BMI**	**Waist/Hip**
**C-Peptide**	1	−0.38 (0.0001)	0.64 (0.0001)	0.45 (0.0001)	0.43 (0.0001)
**IGFBP1**		1	−0.36 (0.0001)	−0.49 (0.0001)	−0.29 (0.0001)
**Insulin**			1	0.45 (0.0001)	0.30 (0.0001)
**BMI**				1	0.35 (0.0001)
**Waist/Hip**					1
		**Men**			
	**C-Pep**	**IGFBP-1**	**Insulin**	**BMI**	**Waist/Hip**
**C-Peptide**	1	−0.28 (0.0001)	0.59 (0.0001)	0.43 (0.0001)	0.41 (0.0001)
**IGFBP1**		1	−0.36 (0.0001)	−0.38 (0.0001)	−0.10 (0.14)
**Insulin**			1	0.42 (0.0001)	0.28 (0.002)
**BMI**				1	0.36 (0.0001)
**Waist/Hip**					1
		**Women**			
	**C-Pep**	**IGFBP-1**	**Insulin**	**BMI**	**Waist/Hip**
**C-Peptide**	1	−0.43 (0.0001)	0.69 (0.0001)	0.46 (0.0001)	0.51 (0.0001)
**IGFBP1**		1	−0.36 (0.0001)	−0.56 (0.0001)	−0.39 (0.0001)
**Insulin**			1	0.48 (0.0001)	0.45 (0.0001)
**BMI**				1	0.43 (0.0001)
**Waist/Hip**					1

## Discussion and conclusion

In this study, we evaluated the relationship between insulin, C-peptide and IGFBP-1 in relation to colorectal adenomas and low apoptosis. We found that elevated levels of insulin and C-peptide predicted increased risk of adenomas in men and women, while elevated levels of IGFBP-1 reduced risk, particularly in men. The risk of adenomas increased with increasing concentrations of C-peptide and insulin, and decreased with increasing concentrations of IGFBP-1. We also found that in overweight and obese individuals higher C-peptide increased the risk of low apoptosis, particularly in men, whereas elevated IGFBP-1 levels were associated with a reduced risk of low apoptosis, an association that was stronger in men. These findings suggest that the mechanism by which these hormones increase risk of adenomas may reflect their anti-apoptotic properties.

Insulin is secreted as pro-insulin and subsequently cleaved into insulin and C-peptide. C-peptide is a marker of pancreatic insulin synthesis, and several epidemiologic studies have utilized C-peptide as an alternate biomarker to insulin because it has a longer half-life than insulin and therefore is more stable [28]. We found positive associations between insulin, C-peptide and adenomas, in both men and women. While these findings contrast with those by Yamaji et al., who found these associations only in men in a Japanese cohort [24], they confirm both our previous observations in an independent patient population [17], and in a recent report in which we found race/ethnicity differences for genes related to the IGF axis [29]. The observed positive association between insulin, C-peptide and adenomas not only highlights the importance of the insulin-IGF pathway in risk of colorectal adenomas and cancer, but it also supports findings from previous studies [19-21,29-31]. Higher IGFBP-1 levels were associated with a reduced risk of adenomas. In these analyses, we did not observe significant differences between cases and controls for IGF-I; these findings are consistent with the hypothesis that higher levels of insulin and C-peptide, and lower levels of IGFBP-1 are the trigger points early in the disease development. Overall, these findings are in agreement with those of others [22], and support the hypothesis that biologically available IGF-1 circulating levels may be an etiologic factor in the formation of adenomas.

Only a limited number of studies have evaluated IGFBP-1 in relation to adenomas or cancer [22,32,33] but the results are inconsistent [19,21,24,29,34]. IGFBP-1, a member of the IGF binding protein family binds to IGF-I or IGF-II to regulate their bioavailability, in this way acting as a modulator of cellular proliferation and cell death [35]. IGFBP-1 can also inhibit metabolism of IGFs [36], and IGFBP-1 expression is negatively regulated by insulin [37]. Levels of circulating C-peptide and insulin inversely correlate with plasma IGFBP-1 levels [14], thus our findings support this observation. They however contrast with a recent report [32] in which high IGFBP-1 levels were inversely associated with colorectal cancer in women, since we only observed statistically significant associations in men. In a larger cohort, Le Marchand et al. also observed a significantly reduced adenoma risk with higher plasma IGFBP-1 levels [22]. Yamaji et al. reported similar associations in Japanese men [24]. Our first attempt to test for IGFBP-1 associations with low apoptosis, showed that overweight and obese individuals with higher C-peptide levels are more likely to have low apoptosis.

Epidemiologic studies suggest that lifestyle and dietary factors play an important role in the etiology of colorectal cancer. In particular, obesity is associated with increased risk of colon cancer, especially in men [38,39]. Men are also more likely to have higher waist-hip ratio, a condition that is associated with elevated levels of insulin and C-peptide [40,41]. Our findings show strong positive correlations between insulin or C-peptide and BMI or WHR among both men and women. In addition, there was a strong inverse association between IGFBP-1 and BMI or WHR. These findings suggest that metabolic characteristics associated with obesity and visceral adiposity may be important biomarkers of adenoma risk.

The major strengths of this study include availability of fasting blood specimens, detailed information on exposures and anthropometrics and ability to measure apoptosis in normal mucosal biopsies and test for correlations with circulating insulin, C-peptide or IGFBP-1 levels. There are some limitations of this study. Plasma samples were only available for one-time measures of insulin, C-peptide and IGFBP-1. However our findings are similar to other studies that used one-time measures of these analytes. Also, future studies should consider measuring insulin resistance based on the Homeostatic Model Assessment (HOMA).

In summary, we found significantly elevated levels of insulin and C-peptide in patients with adenoma when compared to adenoma-free controls. Elevated concentrations of IGFBP-1 reduced the risk for adenomas in men. These findings confirm our previous observations in an independent patient population and support the involvement of the insulin-IGF pathway in the etiology of colorectal carcinogenesis. Elevated C-peptide and insulin significantly predicted adenoma risk although C-peptide appeared a stronger predictor of low apoptosis in overweight and obese men than women. The strong positive association between BMI and WHR, known colorectal cancer risk factors that are also related to hyperinsulinemia and the insulin-IGF axis, suggests an interplay between obesity, insulin-related factors and adenomas. Our findings suggest that the anti-apoptotic properties of these peptide hormones, particularly C-peptide, may be the primary driver of associations with adenomas. Preventive measures to reduce obesity and closer monitoring of individuals may be beneficial to reducing adenoma and cancer risk.

## Competing interests

The authors declared no competing interests.

## Authors’ contributions

TOK, RS and PKL conceived and designed the experiments. OO, LB and RH performed the experiments. TOK, PKL, CH and ACV analyzed and interpreted the data. TOK, ACV and CH wrote the paper. JAG performed statistical analyses. All authors have read and approved the final manuscript.

## Pre-publication history

The pre-publication history for this paper can be accessed here:

http://www.biomedcentral.com/1471-2407/12/389/prepub
